# Experimental conditions influence the formation and composition of the corona around gold nanoparticles

**DOI:** 10.1186/s12645-020-00071-7

**Published:** 2021-01-06

**Authors:** Md. Nazir Hossen, Chandra Kumar Elechalawar, Virginie Sjoelund, Kathleen Moore, Robert Mannel, Resham Bhattacharya, Priyabrata Mukherjee

**Affiliations:** 1 Peggy and Charles Stephenson Cancer Laboratory Research, Oklahoma Stanton L. Young Biomedical Research Center, University of Oklahoma Health Sciences Center, 975 N.E., Suite # 1409 10th Street, Oklahoma City, OK 73104 USA; 2 Department of Obstetrics and Gynecology, University of Oklahoma Health Science Center, Oklahoma City, OK USA; 3 Department of Pathology, University of Oklahoma Health Science Center, Oklahoma City, OK USA; 4 Department of Cell Biology, Mass Spectroscopy/Proteomic Core, University of Oklahoma Health Science Center, Oklahoma City, OK 73104 USA

**Keywords:** Pre-processing conditions, Gold nanoparticles, Protein corona, New molecular target proteins

## Abstract

**Background:**

Ovarian cancer is one of the deadliest gynecological malignancies. While the overall survival of ovarian cancer patients has slightly improved in recent years in the developed world, it remains clinically challenging due to its frequent late diagnosis and the lack of reliable diagnostic and/or prognostic markers. The aim of this study was to identify potential new molecular target proteins (NMTPs) responsible for the poor outcomes. When nanoparticles (NP) are exposed to biological fluids, a protein coat, termed the protein corona (PC), forms around the NP, and the PC represents a tool to identify NMTPs. This study investigates the influence of pre-processing conditions, such as lysis conditions and serum/plasma treatment, on the PC composition and the resulting identification of NMTPs.

**Results:**

Using gel electrophoresis, pre-processing conditions, including cell-lysis techniques and enrichment of low-abundance proteins (LAPs) by immunocentrifugation of serum/plasma, were shown to alter the relative amounts and compositions of proteins. PCs formed when 20 nm gold-NPs (GNPs) were incubated with lysate proteins from either RIPA- or urea lysis. Proteomic analysis of these PCs showed 2–22-fold enrichment of NMTPs in PCs from urea lysates as compared to RIPA lysates. Enriched NMTPs were then classified as cellular components, biological and molecular functions-associated proteins. The impact of enriched LAPs (eLAPs) on both PC composition and NMTP identification was shown by comparative proteomic analysis of original plasma, eLAPs, and PCs derived from eLAPs; eLAPs-PCs enhanced the abundance of NMTPs approximately 13%. Several NMTPs, including gasdermin-B, dermcidin, and kallistatin, were identified by this method demonstrating the potential use of this PC approach for molecular target discovery.

**Conclusion:**

The current study showed that the pre-processing conditions modulate PC composition and can be used to enhance identification of NMTPs.
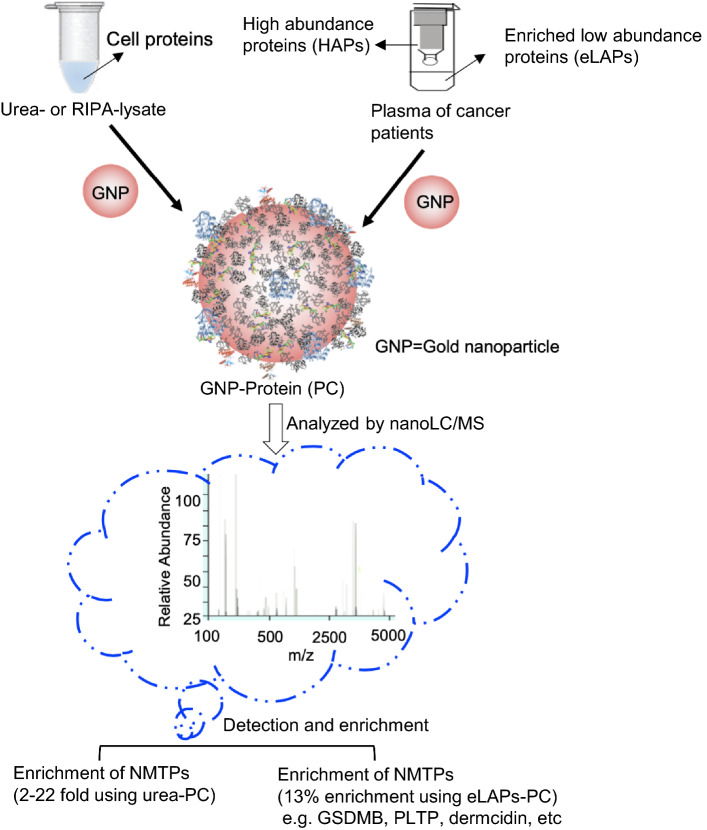

## Background

Ovarian cancer will account for about 21,750 new cases and 13,940 deaths in the United States in 2020 (Siegel et al. 2020). While overall survival for ovarian cancer patients has slightly improved in recent years, it remains a challenging malignancy due to frequent late diagnosis—the lack of reliable diagnostic and/or prognostic markers from patient samples is a major hindrance to improved outcomes. Identification of potentially new molecular targets responsible for poor outcome is the focus of this study; ovarian cancer cell lines and patient plasma samples were investigated as a paradigm for improving the utilization of nanoparticles (NP) to identify proteins of interest (Engelberth et al. 2014).

In 2007, Cedervall et al. coined the term protein corona (PC), to describe the formation of a protein layer around a nanoparticle (NP) in contact with a biological sample (Cedervall et al. 2007). The PC can be divided into two types—a hard PC and a soft PC. In the hard PC, proteins adsorb directly to the NP surface with high affinity; whereas in the soft PC, proteins interact with the hard PC via weak protein–protein interactions (Walkey and Chan 2012). Multiple studies show that the composition of the PC determines the fate of the NP, both in vitro and in vivo, including its cellular uptake, intracellular fate, biodistribution, clearance and toxicity (Lesniak et al. 2012; Nguyen and Lee 2017; Corbo et al. 2016). In addition, analysis of the PC can contribute to the identification of new molecular target proteins (NMTPs) (Elechalawar et al. 2020; Giri et al. 2014; Arvizo et al. 2012). Many factors affect the PC composition; potential factors influencing the PC include characteristics of the NPs (i.e., size, charge, and surface engineering), characteristics of the protein (i.e., molecular weight, isoelectric points, structure and folding), characteristics of the interaction (i.e., time, temperature, concentration), and the type of biological fluid (i.e., plasma, urine, tissue lysate, etc.) (Bohmert et al. 2020; Tenzer et al. 2013; Deng et al. 2009; Vogt et al. 2015; Hadjidemetriou et al. 2015, 2016; Aggarwal et al. 2009). The PC composition is also modulated by the separation technique used to recover NPs from the NP–biological fluid mixture, i.e., centrifugation, ultracentrifugation, magnetism, or chromatography (Bohmert et al. 2020). Similarly, pre-processing conditions may impact the PC composition. Pre-processing variables might include: (1) washing of culture media containing abundant serum proteins causing changes in the number/quantity of proteins available for adsorption by the NP; (2) type of washing buffer influencing protein binding due to changing pH or salt concentration; (3) temperature; or (4) type of lysis buffer affecting the dissolution of cellular or serum/plasma proteins. However, the effect of experimental pre-processing conditions on the modulation of PC compositions has not been carefully investigated to date.

Appropriate modulation of the PC composition is advantageous since it can facilitate the identification of NMTPs for precision diagnosis, prognosis and therapeutic monitoring (Bohmert et al. 2020; Corbo et al. 2017). Modulation can be achieved by optimizing or altering one or more of the parameters described above. For example, optimization of centrifugation time and speed as well as washing steps limited detection of false-positive high-abundance proteins (HAPs) (Bohmert et al. 2020). Another approach to modulate the PC may be to enrich low-abundance proteins (LAPs) in biological fluids prior to processing the fluid with the NP. The proteins in human serum/plasma are approximately 90–95% HAPs (specifically α1-acid glycoprotein, α1-antitrypsin, α2-macroglobulin, albumin, apolipoprotein A-I, apolipoprotein A-II, fibrinogen, haptoglobin, IgA, IgM, IgG, and transferrin) and only 5–10% LAPs (Liumbruno et al. 2010; Thadikkaran et al. 2005). Interference from the HAPs limits the binding of LAPs to NPs and, thus, their detection by subsequent mass spectroscopic methods. This is unfortunate since the LAPs fraction represents a promising source of biomarkers/NMTPs; the LAPs could include proteins resulting from leakage from diseased tissues and/or important cellular ligands and signaling molecules (Millioni et al. 2011). Thus, enriching LAPs in biological fluids prior to their interaction with NPs could have profound benefits for their successful detection and, as a result, identification of promising NMTPs.

**Fig. 1:**
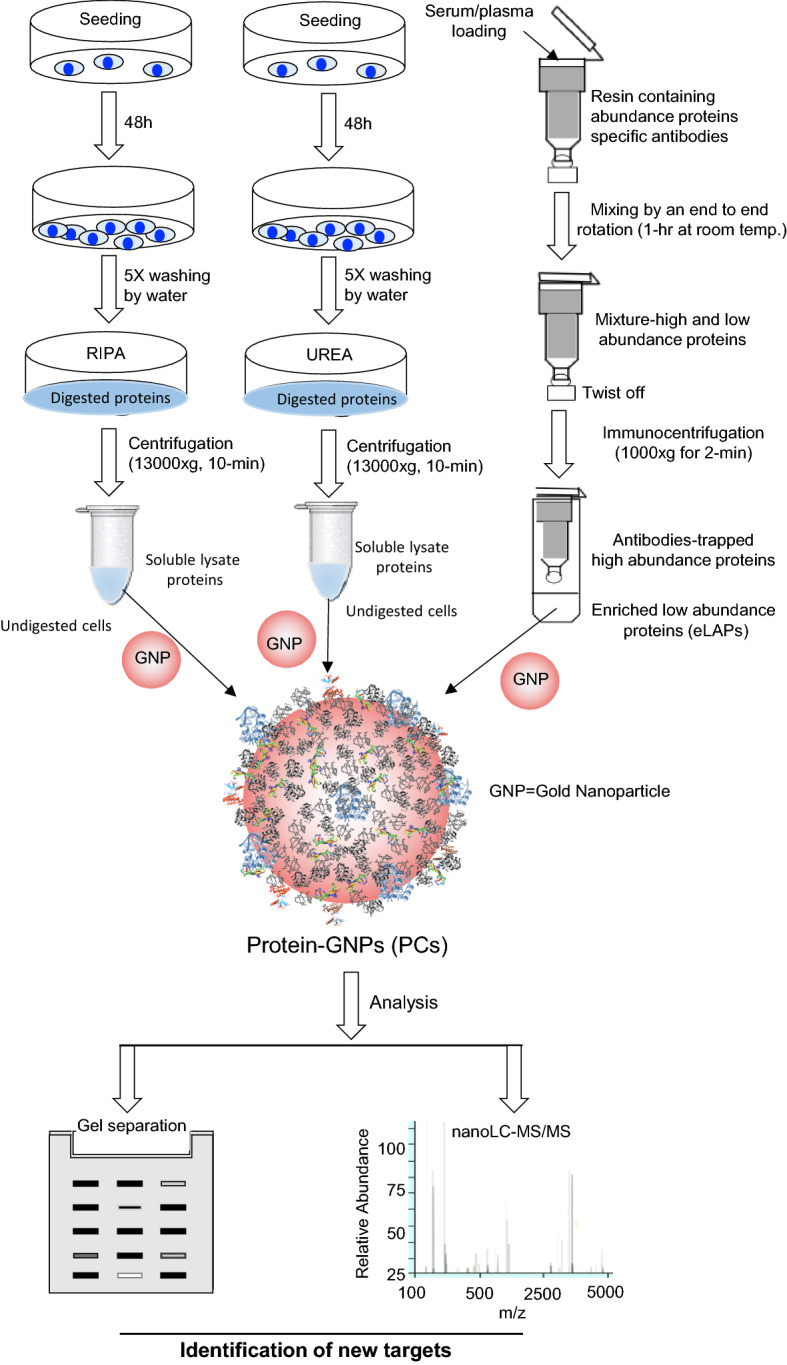
Schematic illustration of work-flow for the preparation, characterization, analysis and identification of new molecular target proteins in the protein corona around gold nanoparticles using cell lysates (RIPA or urea lysis) and low-abundance proteins enriched by immunocentrifugation of serum/plasma

In this study, 20 nm gold-NPs (GNPs) were used as a tool to capture proteins of interest based on their self-therapeutic properties including the ability to bind a number of heparin-binding growth factors (HB-GFs) that may be involved in the proliferation of cancer cells and metastasis. The impact of pre-processing conditions on the formation and composition of the PC around these GNPs was assessed with a view to improving identification of NMTPs (Fig. 1). Lysing ovarian cancer cells with urea, as opposed to the commercial RIPA buffer, increased the abundance of molecular target proteins by 2- to 22-fold. More importantly, when LAPs were enriched in ovarian cancer patient plasma samples by immunocentrifugation the abundance of several NMTPs (e.g., gasdermin-B, dermcidin, and kallistatin) was also increased by around 13%. Thus, altering the conditions under which biological samples are treated prior to exposure to NPs may enhance the identification of NMTPs and facilitate their adoption as biomarkers of disease progression, prognosis and/or treatment success.

## Results and discussion

This study examined the influence of pre-processing conditions on the formation and composition of the PC around GNPs with the ultimate goal of facilitating the identification of new molecular targets (Fig. 1).

### Enrichment of proteins in cell lysates by urea extraction and in serum/plasma by immunocentrifugation

**Fig. 2:**
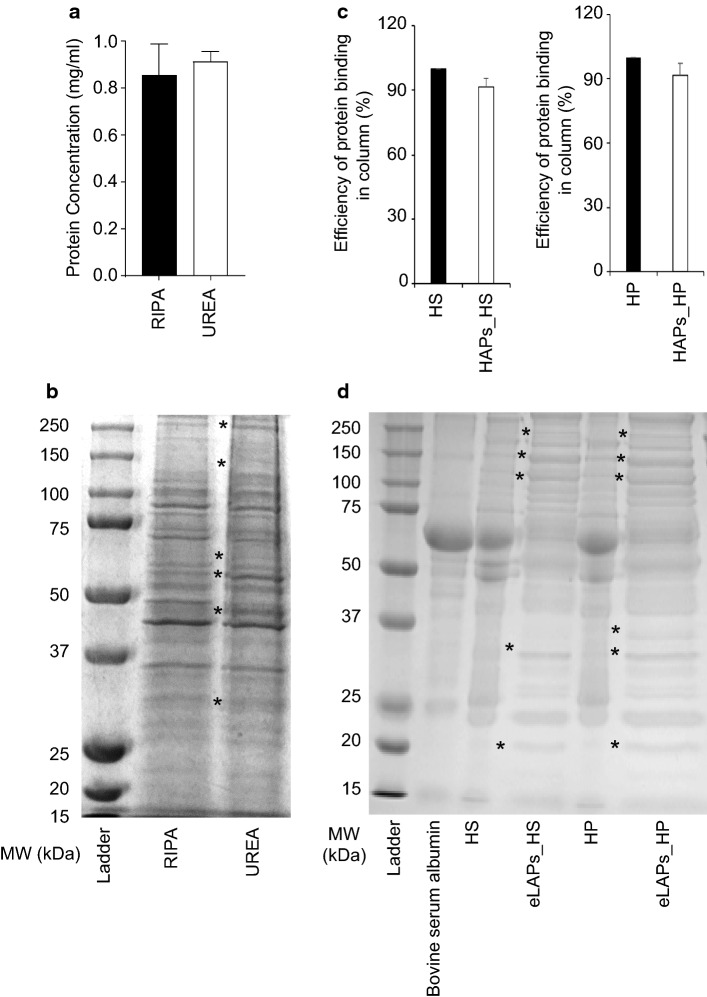
Enrichment of proteins in cell lysates by urea extraction and in serum/plasma by immunocentrifugation. **a**, **c** Measurement of proteins in cell lysates and in serum/plasma using the BCA assay. **a** The lysate proteins of TykNu cells after digestion by RIPA or 8 M urea were measured using the BCA assay. **c** Efficiency of the binding of high-abundance proteins (HAPs) to immuno-affinity columns. HAPs in human plasma (HP) or serum (HS) after column separation were measured by BCA and the efficiency of HAPs binding to the column is shown. **b**, **d** Confirmation of enriched proteins in urea and RIPA lysates and enriched low-abundance proteins (eLAPs) in serum/plasma by gel separation. Denatured peptides/proteins of either RIPA or urea lysates were separated by SDS-PAGE and were visualized by coomassie staining, eLAPs indicates enriched low-abundance proteins and BSA is bovine serum albumin. The asterisk (*) indicates the relative enrichment of peptides/proteins in cell lysates and in depleted serum/plasma, compared to the original serum/plasma

Initially, ovarian cancer cells and ovarian cancer patient plasma/serum samples were used as protein sources for the formation of PC around GNPs. Prior to PC formation, preliminary studies were performed to characterize protein types and levels in cell lysates and plasma samples. To obtain proteins from cancer cells, two different cell lysis buffers were used: (i) the commercial RIPA buffer, and (ii) urea. The protein patterns derived from using the two buffers were compared. Ovarian cancer cells (TykNu) were cultured in media containing 10% fetal bovine serum, lysed with either RIPA or urea and the lysates were analyzed by gel electrophoresis. Equivalent amounts of protein, determined using the BCA method (Fig. 2a), were separated by SDS-PAGE and visualized using Coomassie Blue. There were significant differences in the relative molecular weights of proteins generated by RIPA and urea lysis (Fig. 2b). These data suggest that cell lysis conditions play a critical role in determining the types of protein generated. These results are supported by the report of Marini et al. who showed that when the soluble and insoluble fractions of mitochondria from cancer cells are enzymatically digested by trypsin, Glu-C, or chymotrypsin, each lysis condition produces a distinct protein signature (Marini et al. 2020; Nierenberg et al. 2018).

Human plasma/serum, including the plasma/serum from ovarian cancer patient used herein, generally consists of 90–95% HAPs and 5–10% LAPs. The LAPs fraction represents a potential source of biomarkers/NMTPs. Since HAPs can mask LAPs from detection by mass spectrometry, HAPs are generally depleted by passage through a HAP-specific binding column prior to analysis (Millioni et al. 2011). Thus, HAPs were initially depleted (> 90%) by passage of patient plasma/serum through columns containing immobilized HAP-specific antibodies. The binding efficiency of the HAPs to the columns, for serum and plasma, respectively, was 91.6 ± 4 and 91.7 ± 5.5%, showing that HAPs are efficiently depleted by a single pass through the column (Fig. 2c). The depletion of HAPs was confirmed by SDS-PAGE, and demonstrated the absence of HAP bands across the molecular weight range including bands corresponding to the following specific HAPs: apolipoprotein A-II (17.4 kDa), apolipoprotein A-I (28.3 kDa), haptoglobin (40 kDa), α1-acid glycoprotein (41–43 kDa), α1-antitrypsin (52 kDa), albumin (66.5 kDa), transferrin (80 kDa), and IgA (160 kDa). LAPs bands were enriched in the HAPs-depleted flow through (Fig. 2d). These data confirm that LAPs can be enriched and detected by pre-processing, which would theoretically enhance identification of potential NMTPs.

### Characterization of the protein corona arising from the interaction of gold nanoparticles with cell lysates, plasma, or enriched low-abundance proteins from plasma

**Fig. 3:**
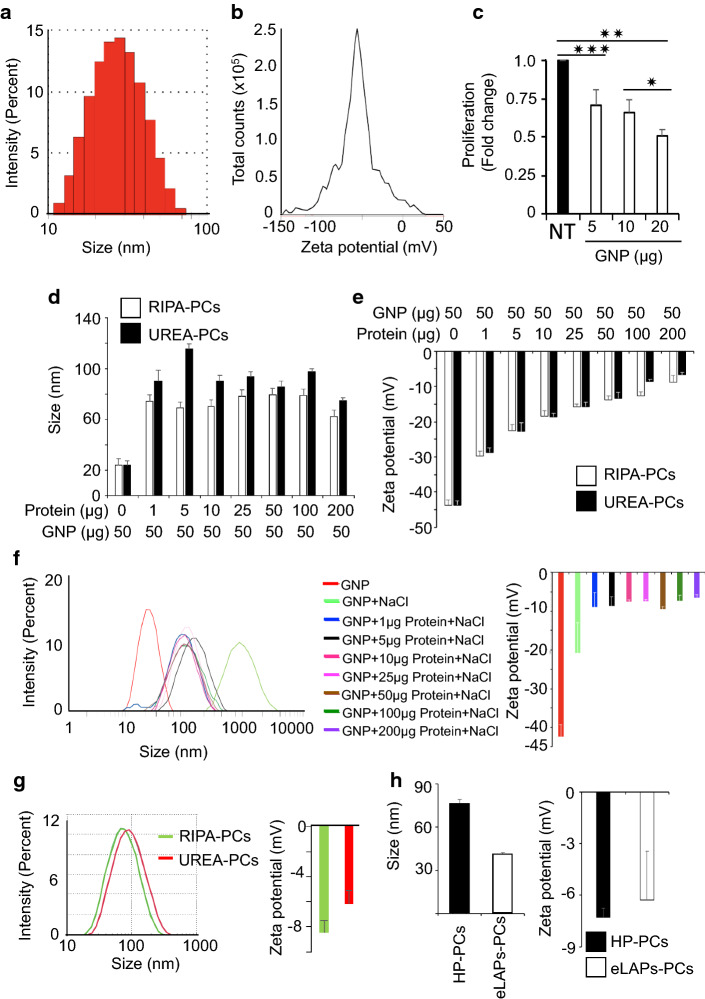
Physicochemical characterizations of protein–gold nanoparticle interactions in urea-lysates or enriched low-abundance proteins (eLAPs) from patient plasma. **a**, **b** The physicochemical characteristics (size and charge) of synthesized GNPs. **c** Biological activity of GNPs in cancer cells. The proliferation of TykNu cells in the presence or absence of GNPs was measured by cyquant assay. Data are expressed as mean ± SD, Student *t-*test, * = P < 0.01, ** = P < 0.001, *** = P < 0.0001, n = 6. **d**, **e** Hydrodynamic diameter (HD) and charges of GNPs after incubation with lysate proteins. The HD and charge of GNPs after incubation with various amounts of urea- and RIPA-lysate proteins were measured using DLS and zeta potential measurements. **f** Stability of GNPs. Protein coronas with 150 mM NaCl were characterized by DLS and zeta potential measurements. **g**, **h** Hydrodynamic diameter (HD) and charge of GNPs after incubation with 200 µg of protein from urea-lysates, human plasma, or eLAPs were characterized by DLS and zeta potential measurements

Next, PCs were formed by the interaction of the various protein preparations with 20 nm GNPs to determine any observable differences in their physicochemical properties, i.e., size and charge. 20 nm GNPs were synthesized by the citrate reduction method (Hossen et al. 2019) and characterized using UV–Visible Spectroscopy (UV–Vis), dynamic light scattering (DLS), ζ-potential measurements, transmission electron microscopy (TEM) and the cyquant proliferation assay. A surface plasmon resonance (SPR) band around 522 nm in the UV–Vis spectrum indicates the formation of spherical ~ 20 nm GNPs (Additional file 1: Fig. S1A–C) (Hossen et al. 2019). The hydrodynamic diameter (HD) and surface charge of GNPs were determined by DLS and zeta potential measurements, respectively; GNPs had an HD of 24.7 ± 3.0 nm and a net negative charge of 42.4 ± 3.2 mV (Fig. 3a, b). TEM confirmed that GNPs of approximately 22.5 ± 3.4 nm in diameter were synthesized by this method (Additional file 1: Fig. S1B, C). Previously, we demonstrated that GNPs possess unique self-therapeutic properties (Arvizo et al. 2013). GNPs bind to a number of heparin-binding growth factors (HB-GFs) at their HB domain and inhibit HB-GF function by altering protein conformation; GNPs of 20 nm had the highest efficacy. This inhibition of protein function resulted in inhibition of tumor growth in both ovarian and pancreatic cancer. Thus, proteins that bind to GNPs may have critical roles in tumor growth and GNPs could be used as a tool to identify such proteins as potential NMTPs. Therefore, before proceeding with NMTP identification, it was important to verify the self-therapeutic property of the GNPs. The self-therapeutic property of GNPs to inhibit proliferation was tested against TykNu ovarian cancer cells. GNPs decreased proliferation of TykNu cells in a dose-dependent manner, as evaluated by the cyquant proliferation assay, indicating that the synthesized GNPs are biologically active (Fig. 3c). This result was consistent with our previous finding that GNP inhibits ovarian cancer cell proliferation (Arvizo et al. 2013; Xiong et al. 2014). Moreover, the findings of others also support the concept that GNPs can be utilized not only to identify therapeutic targets in various diseases, such as diabetic retinopathy, macular degeneration, and rheumatoid arthritis, but also to inhibit angiogenesis in these models (Kim et al. 2011; Tsai et al. 2007).

The synthesized and characterized GNPs were incubated with the RIPA and urea cell lysates to determine if the generated PCs differed in their formation and composition. The two lysates were individually incubated with GNPs at various amounts, and the HD and charge of the resulting PC-coated GNPs were determined. When GNPs were incubated with increasing amounts of the lysates (i.e., 1, 5, 10, 25, 50, 100 and 200 µg), their HD increased (Fig. 3d). Following incubation with the RIPA lysate at the increasing protein amounts, the GNPs had size distributions with z-averages of 74.5 ± 2.1, 69 ± 9.5, 70.5 ± 14.2, 78.3 ± 2.9, 79.7 ± ,1.9, 79 ± 3.8 and 62.2 ± 2.7 nm; for the urea lysate, the sizes were 90.5 ± 8.2, 115.4 ± 3.8, 90.3 ± 4.6, 93.9 ± 3.8, 85.9 ± 4.2, 97.5 ± 2.2 and 74.9 ± 2.0 nm. The increased size of GNPs following incubation with lysate proteins indicates PC formation, and is consistent with the findings of others showing that when biologically active NPs are incubated with biological fluids, they become covered with a protein layer and have an associated increase in size (Brun et al. 2014). In addition, the charge of the NP is a second parameter that indicates whether proteins have bound to the surface. Naked GNPs had a ζ-potential of − 43.6 ± 1.2 mV; the urea lysates alone had charges of -12.7 ± 2.4, − 28.5 ± 3.7, − 31.6 ± 2, − 28.1 ± 5.0, − 31.1 ± 2.7, 25.6 ± 1.46 and − 24.7 ± 2.37 mV; while, the RIPA lysates had charges of − 15.5 ± 2.2, − 33.9 ± 5.9, − 36 ± 9.2, − 31.3 ± 5.6, − 25.4 ± 5.7 and − 25.4 ± 2.7 at the increasing protein amounts, respectively (Additional file 1: Fig S1A–D). Interaction of GNPs with increasing protein amounts of the urea lysate resulted in charges of − 28.7 ± 1.3, − 22.9 ± 2.6, − 18.6 ± 1.0, − 15.7 ± 1.2, − 13.3 ± 1.7, − 8.4 ± 0.5 and  − 6.7 ± 0.7 mV, respectively; while, the corresponding values for GNPs treated with RIPA lysates were − 29.6 ± 1.5, − 23.5 ± 1.7, − 19 ± 1.6, − 15.8 ± 0.9, − 13.3 ± 1.3, − 12.7 ± 1.2 and − 9.6 ± 1.8 mV, respectively (Fig. 3d, e). These results suggest that proteins gradually bound on the surface of GNPs, thereby reducing the charge, and the charge fell more on the addition of increasing protein amounts. Taken together, the increase in size paired with the decreasing charge of GNPs when incubated with lysate proteins demonstrates that the lysate proteins successfully bound on the GNP surface to form a PC.

To further confirm the formation of a PC around the GNPs, aggregation studies were performed by treating the GNP–protein mixture with 150 mM NaCl. Treatment with 150 mM NaCl disrupts the repulsive electrical double layer around unmodified NPs, thereby inducing aggregation. The ability of NaCl to aggregate NPs decreases when the NP surface is coated with proteins (Giri et al. , 2014; Hossen et al. 2020). NaCl was added at a concentration of 150 mM to the formed urea-PCs around GNPs, and the size and surface charge were measured before and after NaCl addition. The ζ-potential of the uncoated GNPs decreased from − 42.4 ± 3.2 to − 20.8 ± 7.8 mV and their size increased from 24.7 ± 3.0 to 584.9 ± 85.0 nm upon the addition of NaCl, indicating their aggregation (Fig. 3f). However, the size of the GNPs with PCs did not significantly change on addition of NaCl (86 ± 2.9, 130.2 ± 10.4, 95.8 ± ,7.5 90.6 ± 2.3, 88.9 ± 5.7, 94.7 ± 14.3 and 78 ± 2.2 nm); in contrast, the surface charge of PCs decreased at the 1–50 µg protein levels (− 9 ± 3.7, − 8.8 ± 2.3, − 7.6 ± 0.5, − 7.5 ± 0.5, − 9.5 ± 0.6 mV) but not at 100 or 200 µg (− 7.4 ± 1.4 and − 6.6 ± 0.8 mV) (Fig. 3f). These aggregation studies demonstrated that there were no appreciable changes in either size or charge following NaCl treatment of the PC-coated GNPs derived from incubation with 50–200 μg of protein, indicating that 50 μg of lysate proteins is the saturation concentration for 1 ml of 20 nm GNP as synthesized.

Similarly, the GNPs incubated in the original plasma samples and the plasma-derived eLAPs had size distributions of z-average 76 ± 3.2 and 41 ± 1.5 nm and ζ-potentials − 7.3 ± 0.5 and − 6.3 ± 2.8 mV, respectively (Fig. 3g, h). The reduced size of the eLAPs incubated GNPs, i.e., 41 versus 76 nm, may result from the attachment of comparatively smaller proteins around GNPs from the eLAPs; the slightly decreased charge indicates the eLAPs probably lead to more stable PCs. Aggregation studies with NaCl showed no appreciable change either in size or charge, suggesting stabilization of the GNP surface by the eLAPs proteins (Fig. 3f). Moreover, it is also likely that a dynamic equilibrium is reached where high affinity proteins are enriched on the GNP surface replacing the low affinity HAPs resulting in decrease in size and charge.

### Impact of cellular protein extraction process on protein corona composition around gold nanoparticle

**Fig. 4:**
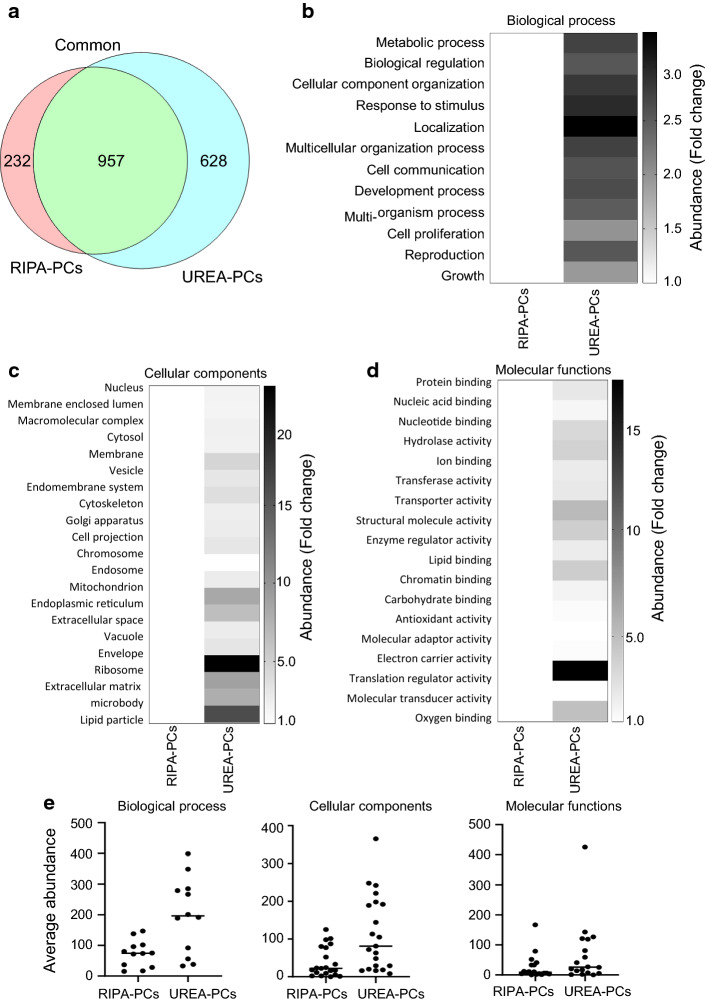
Impact of urea lysates on the composition of the protein corona around gold nanoparticles. **a** Venn diagrams showing proteins identified in the protein corona around GNPs incubated with cell lysates digested by either RIPA or urea. **b**–**d** Heatmaps indicating the abundance of the peptide sequence of identified proteins only in RIPA- and urea lysate. **e** The average enrichment of peptide sequences of identified proteins for each of the above specified categories

Following the confirmation of a stable PC and its physicochemical characterization, the composition of the PCs formed following incubation of GNPs with 200 µg of either urea- or RIPA-lysate proteins was determined using nanoLC–MS/MS. Unbound proteins were removed by centrifugation and GNPs were washed once with water prior to analysis, and equal amounts of bound protein were trypsin-digested for analysis by nanoLC–MS/MS. Nine-hundred and fifty seven common proteins were identified, 232 proteins were found in only the RIPA-derived PCs and 628 proteins in only the urea-lysate PCs. The details of the identified proteins are provided in Additional file 2: Tables S1 and Additional file 3: Table S2. Many of the proteins were significantly enriched (2- to 22-fold) in the urea sample compared to RIPA (Fig. 4a–c). The common proteins (957) are most likely abundant proteins and served as a detectable lysate pool to compare the differential properties of the attached proteins and to assess the enrichment of proteins on the GNP surface. In addition to these common lysate proteins, the LAPs extracted by RIPA and urea are bound on the GNP surface according to their affinities. These LAPs were classified into three gene ontology (GO) categories: cellular components, biological functions, and molecular functions (Fig. 4b–d and Additional file 1: Fig. S2). Urea-lysate proteins for biological processes were mainly related to localization, stimulus, metabolic processes and reproduction; whereas among the cellular components, envelope, microbody, mitochondria, and membrane were most abundant. In addition, electron carrier activity is a major pathway related to molecular function (Fig. 4b–d and Additional file 1: Fig. S2). The average abundance of the peptides from these proteins was higher in the urea-lysates than in the RIPA (Fig. 4e). Thus, cellular protein extraction using urea may increase the abundance of NMTPs.

### Identification of new molecular target proteins in the protein corona around gold nanoparticle from enriched low-abundance proteins

**Fig. 5:**
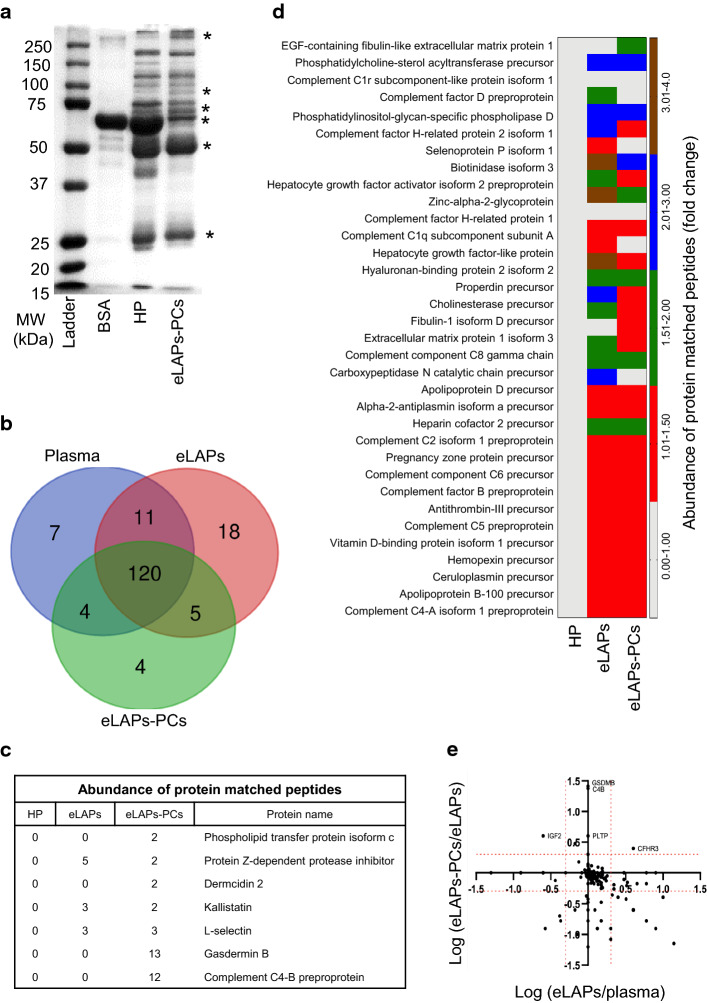
Identification of new molecular target proteins in the protein corona around gold nanoparticles from enriched low-abundance proteins (eLAPs). **a** Gel separation of proteins around GNPs. Denatured proteins/peptides in human plasma (HP) or eLAPs were separated by gel electrophoresis and visualized. The asterisk (*) indicates the relative enrichment of low-abundance peptides/proteins in plasma, compared to the original plasma. **b** Venn diagrams depicting proteins identified by proteomic analysis of both eLAPs and the protein coronas around GNPs incubated with either HP or eLAPs **c**, **d** Heatmap and table showing the enrichment of some important proteins in HP, eLAPs and eLAPs-PCs that were identified by nanoLC–MS/MS. **e** The log of the concentration ratio of eLAPs-PCs to eLAPs and eLAPs to HP. GSDMB, C4-B, PLTP c, DCD 2, IGF2 and CFHR3 denote Gasdermin B, Complement C4-B, Phospholipid transfer protein isoform c, Dermcidin (DCD), Insulin-like growth factor 2 and complement factor H-related protein 3, respectively. The red dotted line delineates a fold change over 2 (considered significant)

To analyze PC composition, plasma from ovarian cancer patients was selected in preference to serum since plasma proteins are more stable than serum proteins. Initially, the relative amount of protein in human plasma (HP), the HP-derived PCs, and the eLAPs-derived PCs was assessed by SDS-PAGE. The PCs from eLAPs showed enrichment of various proteins across the molecular weight range (Fig. 5a and Additional file 1: Fig. S3). NanoLC–MS/MS was used to characterize the proteins in eLAPs, and in the PC of GNPs incubated in either human plasma or eLAPs. A total of 170 proteins were identified; 18 new proteins were enriched in eLAPs, and an additional four proteins were identified in the PC around GNPs incubated with eLAPs. The four proteins identified in eLAPs-PCs were gasdermin-B, dermcidin 2, phospholipid transfer protein isoform c and complement C4-B preproprotein, and their presence suggests enrichment of specific proteins on the surface of GNPs. The detailed list of the identified proteins is provided in the supplementary data (Additional file 4: Table S3). The differences in the protein composition of the various samples are shown in the Venn diagram in Fig. 5b, and most of the promising proteins for future clinical application are shown in Fig. 5c. In addition, the heatmap directly compares the protein composition of the samples in terms of fold change of abundance of protein matched peptides (Fig. 5d). To avoid a bias towards the ratio greater than 1, the log of the concentration ratio is shown and allows the data of ratio less than 1 to be compressed between 0 and 1, while the data for fold changes are symmetric (Fig. 5e). Taken together, these data demonstrate that pre-processing of human plasma by immunocentrifugation, followed by incubation with GNPs to generate a PC, will allow enrichment of LAPs by approximately 13%, thus facilitating the identification of NMTPs that are either undetectable or at very low abundance in ovarian cancer patients’ plasma.

Proteins that were enriched in the PC around GNPs incubated in eLAPs included gasdermin-B (47 kDa), dermcidin isomer 2 (16 kDa), kallistatin (58 kDa), phospholipid transfer protein isoform c (80 kDa), EGF-containing fibulin-like extracellular matrix protein 1 (55 kDa), and selenoprotein p isoform 1 (50–60 kDa) (Fig. 5b-f and Additional file 1: Fig. S4). All of these proteins are involved in tumorigenesis (Hergueta-Redondo et al. 2014; Bancovik et al. 2015; Albers et al. 2012; Zhou et al. 1992; Gao et al. 2010; Wang et al. 2020; Han et al. 2017; Seeliger et al. 2009; Chao et al. 2017). Proteomic analysis confirmed that these proteins were not detected in the original plasma samples, possibly due to their low abundance. Since GNPs have high affinities for specific proteins, the eLAPs may concentrate around the surface of GNPs. For example, gasdermin-B, dermcidin isomer 2 and phospholipid transfer protein isoform c were undetectable in either the original plasma or the eLAPs themselves, but they were found in the PC derived from eLAPs.

Gasdermin-B is a 47-kDa protein which is expressed in several tumor types, including hepatocarcinoma, and gastric, cervical and breast cancers, and its over-expression is related to tumor progression (Hergueta-Redondo et al. 2014). Over-expression of dermcidin isomer 2, a 11-kDa protein, occurs in multiple human tumor types, including melanoma, cutaneous tumors, and breast, prostate, pancreatic, and hepatocellular carcinomas (Bancovik et al. 2015). Phospholipid transfer protein isoform c (PLTP) is associated with lipoprotein metabolism and lipid transport in the vascular compartment (Albers et al. 2012). Kallistatin is a serine proteinase inhibitor (serpin) that has diverse functions in apoptosis, inflammation and tumorigenesis (Zhou et al. 1992; Gao et al. 2010) and EGF-containing fibulin-like extracellular matrix protein 1 is a potential biomarker for diagnosis, prognosis and therapeutic assessment in osteosarcoma, glioma, bladder cancer, human pancreatic adenocarcinoma and pleural mesothelioma (Wang et al. 2020; Han et al. 2017; Seeliger et al. 2009). In addition, the contribution of selenoprotein p isoform 1 (SELENOP) to tumor formation and progression has recently been established (Chao et al. 2017). Most of these proteins identified by the proteomic analysis of PCs have not been studied in ovarian cancer. Thus, these findings create an exciting avenue of further study to validate and apply the identified NMTPs to the management of ovarian cancer.

## Conclusion

This study reveals the influences of experimental conditions on the formation and composition of the PC around 20 nm GNPs; the findings from this study may facilitate the identification of new therapeutic targets in cancer. Both ovarian cancer cell lines and ovarian cancer patient plasma samples were used as protein sources to form a PC around GNPs. To obtain proteins from ovarian cancer cells, two types of cell lysis buffers were used: (i) RIPA and (ii) urea. SDS-PAGE was used to characterize the PC formed around GNPs by the two different cell lysates. There were significant differences in the relative molecular weights of proteins derived from the RIPA and urea lysates (Fig. 2b). These data suggest that cell-lysis conditions play a critical role in determining the types of protein generated. These results are supported by the report of Marini et al., who showed that when the soluble and insoluble fractions of mitochondria from cancer cells were enzymatically digested by trypsin, Glu-C, or chymotrypsin, each enzyme produced a distinct protein signature (Marini et al. 2020; Nierenberg t al. 2018).

In general, human plasma, in this study specifically plasma from ovarian cancer patients, consists of 90–95% HAPs and 5–10% LAPs. The LAPs fraction represents a potential source of novel biomarkers/NMTPs. Since HAPs can mask LAPs from detection by mass spectrometry, HAPs are generally depleted by passage through a HAP-specific binding column prior to mass spectrometry (Millioni et al. 2011). Therefore, herein, HAPs were initially depleted (> 90%) by passage of patient plasma through a HAP-specific column and the depletion confirmed by SDS-PAGE; stained gels confirmed the absence of HAP bands in the molecular range indicative of apolipoprotein A-II, apolipoprotein A-I, haptoglobin, α1-acid glycoprotein, α1-antitrypsin, albumin, and transferrin among others. Importantly, the LAP bands were enriched in the flow through (Fig. 2d). Cell lysate proteins and column-passed plasma (i.e., enriched LAPs; eLAPs) were used to study PC formation by incubating either increasing amounts of lysate proteins (1–200 µg) or 200 µg of eLAPs with a fixed amount of GNPs. Formation of PCs were confirmed by the increase in hydrodynamic diameter (HD) and decrease in charge of GNPs as measured by DLS and zeta potential, respectively. This is supported by published data demonstrating that when NPs are incubated with biological fluids, a PC forms around the NP with an associated increase in size (Brun et al. 2014). To further confirm PC formation around the GNPs, aggregation studies were performed by treating the GNP–protein mixture with 150 mM NaCl. Treatment with 150 mM NaCl disrupts the repulsive electrical double layer around unmodified NPs, thereby inducing aggregation. However, the ability of NaCl to aggregate NPs decreases as increasing amounts of protein associate with the NP surface (Hossen et al. 2020; Giri et al. 2014). These aggregation studies showed no appreciable changes to either HD or charge (zeta potential) following NaCl treatment of the GNPs mixed with protein in the range of 50–200 μg. This indicated that 50 μg of lysate proteins saturates 1 ml of the 20 nm GNPs as synthesized obtained (Fig. 3f).

Having confirmed PC formation, the protein compositions of the PCs were determined by nanoLC–MS/MS. This revealed a 2–22-fold enrichment of NMTPs in the PCs derived from urea lysates, compared the PCs from RIPA lysates (Fig. 4). It has previously been shown that urea predominantly solubilizes the cytoskeleton proteins of the cell membrane; whereas, RIPA preferentially solubilizes intracellular proteins including those from nucleus, cytoplasm, mitochondria (Ngoka 2008; Grover et al. 1985). Since the nature of the proteins produced by RIPA and urea lysis differs, it is not surprising that the composition of the PC formed using these lysates also differs. Importantly, proteomic analysis to determine the composition of eLAPs-derived PCs found that the abundance of NMTPs was approximately 13% greater in the eLAPs-PCs than in the original human plasma or the eLAPs themselves. Several specific NMTPs were identified in the eLAPs-PCs, including gasdermin-B, dermcidin, and kallistatin (Fig. 5). These results demonstrate the utility of enriching LAPs using the PC approach as a means to identify potential NMTPs in cancer. The current study highlights the importance of experimental conditions for PC formation around GNPs and presents a unique way to identify possible molecular targets in cancer.

## Materials and methods

### Synthesis and characterization of 20 nm gold nanoparticles

20 nm GNPs were prepared as previously described (Hossen et al. 2019). Briefly, 10 mM gold III chloride trihydrate solution (cat. 520918, Sigma-Aldrich, St. Louis, MO, USA) was diluted 40 times with endotoxin-free water and heated to boiling. Prewarmed 1% sodium citrate tribasic trihydrate solution was added and the solution maintained at for 10–15 min until the solution becomes dark purple. The solution was transferred to room temperature and stirred overnight. Synthesized GNPs were characterized by UV–Visible spectroscopy (Spectrostar Nano, BMG Labtech), dynamic light scattering (DLS), zeta potential measurement (Malvern Zetasizer Nano ZS) and transmission electron microscopy (TEM) (Hitachi H7600 Transmission Electron Microscope). UV–Vis and TEM observation of GNPs were performed by previously described methods (Hossen et al. 2019).

### Cell culture and lysates preparation

Ovarian cancer TykNu cells were obtained from the Japanese Collection of Research Bioresources Cell. Cells were cultured in EMEM media supplemented with 10% heat-inactivated FBS (Gibco, Grand Island, NY, USA) and 100 units penicillin and 100 µg streptomycin/ml (Invitrogen, Rockford, IL, USA) in a 5% CO2-humidified atmosphere. Cells from 70 to 80% confluence dishes were used to prepare cell lysates with RIPA (#BP-115, Boston Bioproducts, Ashland, MA, USA) or 8 M urea (cat. U5378 Sigma-Aldrich, St. Louis, MO, USA) buffer containing the protease–phosphatase mix (Thermo Scientific, Grand Island, NY 14072 USA) according to the protocols.

### Protein quantification

The protein quantification in cell lysates, human serum or human plasma, or in HAP-depleted serum or plasma was performed using the BCA protein assay according to the manufacturer’s protocol (Pierce BCA protein assay kit, cat. 23250, Thermoscientific, Grand Island, NY 14072 USA). In brief, each standard (BSA) at known concentrations ranging from 0.125–2 mg/mL, and every sample were loaded into a separate well of a clear, flat-bottomed 96-well microplate. 10 µL volumes of all standards and samples were tested in triplicate. 80 µL of BCA working reagent (A + B) was added to each well and the plate was incubated for at least 30 min at 37 °C. After cooling to room temperature, the absorbance of all samples and standards was measured at 562 nm on a CLARIOstar plate reader (BMG Labtech, Ortenberg, Germany) to determine protein concentration.

### Proliferation assay

Ovarian cancer TykNu cells were plated into a 96-well plate in the presence of serum containing media. After 24 h, cells were treated with various doses (5, 10 and 20 µg) of GNPs. After 48 h, cells were washed with PBS (× 3) and cell proliferation was determined using the CyQUANT NF Cell Proliferation Assay Kit (Invitrogen, C7026) according to the manufacturer’s protocol and the fluorescence intensity was measured at excitation at 485 nm and emission detection at 530 nm.

### Depletion of high-abundance proteins

Depletion of high-abundance proteins from ovarian cancer patient serum or plasma was performed using Pierce™ Top 12 Abundant Protein Depletion Spin Columns according to the manufacturer’s protocol (cat. 85165). Briefly, 10 µl of serum or plasma was loaded into the depletion spin column after equilibration of the column to room temperature. Then, the mixture containing serum or plasma and abundance protein specific antibodies in resin in the column was incubated at room temperature on an end-over-end rotator for 60 min. The bottom closure of the column was removed, and the filtrate was collected into a 1.5 ml tube by centrifugation at 1000x*g* for 2 min. The protein concentration in the original plasma or serum and in the depleted plasma or serum was measured using the BCA assay; subtraction of the concentration of the enriched low-abundance protein serum or plasma from the concentration of original serum or plasma was used to calculate the binding efficiency of HAP to the column.

### Determination of saturating protein amount for protein corona

Protein coronas were formed by mixing various amounts (1, 5, 10, 25, 50, 100, and 200 µg) of lysate proteins with GNPs for 18 h with end-to-end rotation at 4 °C. The size and charge of PCs were determined by DLS and zeta potential measurements. To identify the saturated protein amounts, we added 150 mM NaCl solution to each PC preparation or to GNPs alone and mixed for 15 min. The size and charge of PCs and GNP after addition of NaCl were also determined by DLS and zeta potential measurements. We also prepared PCs around GNPs using serum or plasma or depleted serum or plasma (200 µg) by the above procedure.

### Gel electrophoresis

The original serum or plasma and HAP-depleted serum or plasma were characterized by 1D gel electrophoresis. Each sample was digested by Laemmli buffer containing 1% w/v SDS and 10% 2-mercaptoethanol (v/v) and 15.0 µg of sample was loaded on 10% SDS-PAGE gels. After electrophoresis, gels were washed by ddH_2_O three times for 5 min to remove SDS which interferes with the staining. Water was completely removed from the gel container and coomassie stain was added (cat. 1,610,435, Bio-Rad, Hercules, California, USA). The staining tray was gently shaken for 1 h and then the gel was rinsed in 200 ml of ddH_2_O for at least 30 min, or until the gel had sufficiently destained. Destained gels were photographed using a digital camera.

### Sample preparation and its identification by mass spectroscopy (LC–MS/MS)

The PCs (proteins bound to GNPs) were centrifuged at 10000xg for 20 min and the pellets were washed with ddH_2_O. The final concentration of proteins bound on GNPs was measured by the BCA method and equal amounts of protein from each sample were trypsin-digested as described previously (Giri et al. 2014; Arvizo et al. 2012; Wisniewski et al. 2009). Briefly, proteins in cell lysates (RIPA and UREA lysates) or human plasma or enriched low-abundance proteins (eLAPs) or eLAPs-PCs were trypsin-digested and were then reduced with 10 mM DTT (dithiothreitol) and then alkylated with 10 mM iodoacetamide. The cell lysate was then digested overnight at 37 °C with a 1:20 protein to trypsin ratio in 10 mM ammonium acetate. The peptides were eluted in 10 mM ammonium acetate pH 8.0, desalted using a C18 spin column according to the manufacturer’s protocol (ThermoFisher, CA), dried and resuspended in 10 mM ammonium formate pH 10.0. All nanoparticles are removed either in the filtration step in the 10 MWCO amicon (FASP) or during the C18 clean-up. Liquid chromatography–tandem mass spectrometry was performed by coupling a nanoAcquity UPLC (Waters Corp., Manchester, UK) to a Q-TOF SYNAPT G2S instrument (Waters Corp., Manchester, UK). Each protein digest (about 100 ng of peptide) was delivered to a trap column (300 µm × 50 mm nanoAcquity UPLC NanoEase Column 5 µm BEH C18, Waters Corp, Manchester, UK) at a flow rate of 2 µl/min in 99.9% solvent A (10 mM ammonium formate pH 10, in HPLC grade water). After 3 min of loading and washing, peptides were transferred to another trap column (180 µm × 20 nanoAcquity UPLC 2G-V/MTrap 5 µm Symmetry C18, Waters Corp, Manchester, UK) using a gradient from 1 to 60% solvent B (100% acetonitrile). The peptides were then eluted and separated at a flow rate of 200 nL/min using a gradient from 1 to 40% solvent B (0.1% FA in acetonitrile) for 60 min on an analytical column (7.5 µm × 150 mm nanoAcquity UPLC 1.8 µm HSST3, Waters Corp, Manchester, UK). The eluent was sprayed via PicoTip Emitters (Waters Corp, Manchester, UK) at a spray voltage of 3.0 kV and a sampling cone voltage of 30 V and a source offset of 60 V. The source temperature was set to 70 °C. The cone gas flow was turned off, the nano flow gas pressure was set at 0.3 bar and the purge gas flow was set at 750 ml/h. The SYNAPT G2S instrument was operated in data-independent mode with ion mobility (HDMSe). Full scan MS and MS2 spectra (m/z 50—2000) were acquired in resolution mode (20,000 resolution FWHM at m/z 400). Tandem mass spectra were generated in the trapping region of the ion mobility cell by using a collisional energy ramp from 20 V (low mass, start/end) to 35 V (high mass, start/end). A variable IMS wave velocity was used. Wave velocity was ramped from 300 to 600 m/s (start to end) and the ramp was applied over the full IMS cycle. A manual release time of 500 µs was set for the mobility trapping and a trap height of 15 V with an extract height of 0 V. The pusher/ion mobility synchronization for the HDMSe method was performed using MassLynx V4.1 and DriftScope v2.4. LockSpray of Glufibrinopeptide-B (*m*/*z* 785.8427) was acquired every 60 s and lock mass correction was applied post acquisition. The proteins were identified by bioinformatics using the PLGS (protein Lynx Global Software, Waters) using the human proteome and a reverse mock database. The results were filtered for 90% confidence. The structure of these proteins is not known unless their structures are available in the protein data bank (PDB).

### Statistical analysis

All numerical results are reported as mean ± s.d. The data were presented here from a minimum of 2–3 independent experiments unless stated. The statistical significance of the difference between groups was performed using student *t*-test via prism pad software.

## Supplementary Information

**Additional file 1: **Supplementary figures.

**Additional file 2: Table S1. **Identified proteins by nanoLC-MS/MS in RIPA-PC.

**Additional file 3: Table S2. **Identified proteins by nanoLC-MS/MS in UREA-PC.

**Additional file 4: Table S3. **Identified proteins by nanoLC-MS/MS in original plasma, eLAPs and eLAPs-PC.

## Data Availability

Not applicable.

## Abbreviations

NP: Nanoparticle
PC: Protein corona
NMTPs: New molecular target proteins
eLAPs: Enrichment of low abundance proteins
RIPA: Radioimmunoprecipitation assay
HAPs: High abundance proteins
LAPs: Low abundance proteins
HP: Human plasma
GNPs: Gold nanoparticles
BCA: Bicinchoninic acid assay
BSA: Bovine serum albumin
SDS: Sodium dodecyl sulfate
PAGE: Polyacrylamide gel
DLS: Dynamic light scattering
UV–Vis: Ultraviolet–Visible spectroscopy
TEM: Transmission electron microscopy
SRP: Surface plasmon resonance
HD: Hydrodynamic diameter
mV: Millivolt
nm: Nanometer
NaCl: Sodium chloride
mM: Millimolar
µg: Microgram
ζ-potentials: Zeta potentials
PLTP: Phospholipid transfer protein isoform c
EGF: Epidermal growth factor
SELENOP: Selenoprotein p isoform 1
FBS: Fetal bovine serum
µL: Microliter
mg/mL: Milligram per milliliter
°C: Degrees Celsius
mins: Minutes
h: Hour
PBS: Phosphate buffer saline
g: Gravity
1-D: One-dimensional
ddH_2_O: Double distilled water
DTT: Dithiothreitol
MWCO: Molecular weight cut-off
UPLC: Ultra performance liquid chromatography
HPLC: High performance liquid chromatography
FA: Formic acid
V: Volt
MS: Mass Spectrum
GSDMB: Gasdermin B
C4-B: Complement C4-B
DCD 2: Dermcidin 2
IGF2: Insulin like growth factor 2
CFHR3: Complement factor H-related protein 3
