# Supplementary material for: Experimental conditions influence the formation and composition of the corona around gold nanoparticles
Source: Cancer Nanotechnol. 2021 Jan 6;12(1):1. doi: 10.1186/s12645-020-00071-7 (PMC7788026; doi:10.1186/s12645-020-00071-7)
Supplement: Supplementary file 1 — Additional file 1: Supplementary figures. [file 12645_2020_71_MOESM1_ESM.docx]

**Supplementary Information for**

**Experimental Conditions Influence the Formation and Composition of the Corona Around Gold Nanoparticles**

Md. Nazir Hossen^1,3^, Chandra Kumar Elechalawar^1,3^, Virginie Sjoelund^4^, Kathleen Moore^2^, Robert Mannel^2^, Resham Bhattacharya^2^ and Priyabrata Mukherjee ^1,3^*

**Abstract**

Background: Ovarian cancer is one of the deadliest gynecological malignancies. While the overall survival of ovarian cancer patients has slightly improved in recent years in the developed world, it remains clinically challenging due to its frequent late diagnosis and the lack of reliable diagnostic and/or prognostic markers. The aim of this study was to identify potential new molecular target proteins (NMTPs) responsible for the poor outcomes. When nanoparticles (NP) are exposed to biological fluids, a protein coat, termed the protein corona (PC), forms around the NP, and the PC represents a tool to identify NMTPs. This study investigates the influence of preprocessing conditions, such as lysis conditions and serum/plasma treatment, on the PC composition and the resulting identification of NMTPs.

Results: Using gel electrophoresis, preprocessing conditions, including cell-lysis techniques and enrichment of low-abundance proteins (LAPs) by immunocentrifugation of serum/plasma, were shown to alter the relative amounts and compositions of proteins. PCs formed when 20 nm gold-NPs (GNPs) were incubated with lysate proteins from either RIPA- or urea-lysis. Proteomic analysis of these PCs showed 2-22-fold enrichment of NMTPs in PCs from urea lysates as compared RIPA lysates. Enriched NMTPs were then classified as cellular components, biological and molecular functions-associated proteins. The impact of enriched- LAPs (eLAPs) on both PC composition and NMTP identification was shown by comparative proteomic analysis of original plasma, eLAPs, and PCs derived from eLAPs; eLAPs-PCs enhanced the abundance of NMTPs approximately 13%. Several NMTPs, including gasdermin-B, dermcidin, and kallistatin, were identified by this method demonstrating the potential use of this PC approach for molecular target discovery.

Conclusion: The current study showed that the preprocessing conditions modulate PC composition and can be used to enhance identification of NMTPs.

**Keywords:** Preprocessing conditions, Gold nanoparticles, Protein corona, New molecular target proteins

**Figure S1**. Characterizations of 20nm Gold Nanoparticles by using UV-Vis spectroscopy and transmission electron microscopy. (A) UV-Vis spectrum of as synthesized 20nm gold nanoparticles. (B) TEM micrograph of 20 nm gold nanoparticles. Scale bar 100nm. (C) Size distribution of GNPs by DLS (D) Charges of urea and RIPA lysates that were measured by DLS.

**Figure S2**. Categories of identified proteins in RIPA- and UREA-PC lysates. The bar graphs display the identified proteins only in RIPA, UREA and common in RIPA_UREA around GNPs, that are associated with biological process, cellular components and molecular functions categories.

**Figure S3**. Gel separation of plasma proteins around GNP. The protein corona of HP and eLAPs from HP were formed around GNP and the enriched proteins/peptides around GNPs were separated by SDS-PAGE gel electrophoresis. The peptides/proteins in gel were visualized by coomassie staining. The asterisk (*) symbol indicates a comparison of PCs compositions from original plasma and enriched LAPs

**Figure S4**. Global and PC-related enrichment of plasma proteins. (A) Enrichment of proteins by the effect of only GNP in terms of log concentration. (B) Global enrichment of proteins in eLAPs over plasma and in eLAPs-PC over eLAPs were expressed by log concentration.
